# The *qSAC3* locus from *indica* rice effectively increases amylose content under a variety of conditions

**DOI:** 10.1186/s12870-019-1860-5

**Published:** 2019-06-24

**Authors:** Hua Zhang, Lihui Zhou, Heng Xu, Liangchao Wang, Huijie Liu, Changquan Zhang, Qianfeng Li, Minghong Gu, Cailin Wang, Qiaoquan Liu, Ying Zhu

**Affiliations:** 1 State Key Laboratory Breeding Base for Zhejiang Sustainable Pest and Disease Control, Key Laboratory of Creative Agriculture, Ministry of Agriculture, Institute of Virology and Biotechnology, Zhejiang Academy of Agricultural Sciences, Zhejiang, 310021 Hangzhou China; 2 Jiangsu High Quality Rice Research and Development Center, Nanjing Branch of China National Center for Rice Improvement, Provincial Key Laboratory of Agrobiology, Institute of Food Crops, Jiangsu Academy of Agricultural Sciences, Nanjing, 210014 Jiangsu China; 3 Key Laboratory of Plant Functional Genomics of the Ministry of Education, Jiangsu Key Laboratory of Crop Genetics and Physiology, College of Agriculture, Yangzhou University, Yangzhou, 225009 Jiangsu China

**Keywords:** Rice quality, Chromosome segment substitution lines, Amylose content, Quantitative trait loci, Environmental effect

## Abstract

**Background:**

Amylose content (AC) is a critical factor for the quality of rice. It is determined by the biosynthesis gene *Waxy* (*Wx*) and a variety of quantitative trait loci (QTLs). Although many QTLs have been reported to affect rice AC, few of them have been investigated under varying growth conditions, especially various temperatures, which are known to greatly influence the AC.

**Results:**

We analyzed the AC at different temperatures and planting seasons in a set of chromosome segment substitution lines (CSSLs) which were derived from a cross between the *indica* variety 9311 and the *japonica* variety Nipponbare carrying the same *Wx*^*b*^ allele. A joint analysis detected a single locus, *qSAC3*, with a high logarithm of odds (LOD) score in four different conditions. The *qSAC3* from *indica* 9311 (*qSAC3*^*ind*^) substantially increased the AC in *japonica* Nipponbare under all tested growth conditions. Furthermore, introducing the *qSAC3*^*ind*^ into the soft rice variety Nangeng9108 with *Wx*^*mq*^, a mutant allele of *Wx*^*b*^, also moderately increased its AC and improved its appearance quality significantly by reducing the chalkiness of the polished rice.

**Conclusions:**

Our results indicate that the *qSAC3*^*ind*^ could increase the AC of *japonica* rice in different environments as well as in the background of different *Wx* alleles and that *qSAC3* is a valuable locus for fine-tuning the rice AC and ameliorating the dull endosperm in rice varieties with the *Wx*^*mq*^ allele.

**Electronic supplementary material:**

The online version of this article (10.1186/s12870-019-1860-5) contains supplementary material, which is available to authorized users.

## Background

Rice is a major cereal crop feeding more than half of the global population. As economies develop, the demand for high-quality rice is increasing. Rice quality covers many aspects, such as milling quality, grain appearance, and eating and cooking quality. Among them, eating and cooking quality receive the most attention. Three physicochemical properties, the amylose content (AC), gel consistency (GC) and gelatinization temperature (GT), determine rice eating and cooking quality, while the AC is regarded as the most important [1]. Consumers from different areas prefer different tastes of rice which is associated with different AC. Unraveling the regulatory mechanism of AC determination is indispensable to breeding rice with desired taste to meet the different demands. The AC is not only determined by genetic factors but is also affected by environmental conditions [2–5].

It is well documented that AC is controlled by one major locus and many minor quantitative trait loci (QTLs) in the rice genome. The major locus, *Wx*, is located on chromosome 6 [4–7]. It encodes the GBSSI (granule-bound starch synthase I) protein, which is mainly responsible for the amylose synthesis in endosperm and pollen sac [8]. Two major *Wx* alleles, *Wx*^*a*^ and *Wx*^*b*^, occur widely in Asian cultivated rice (*Oryza sativa L*). Between them, a single nucleotide polymorphism (G to T) was found in the splicing site of the first intron, which affects the splicing efficiency of *Wx* pre-mRNA [9, 10]. *Wx*^*a*^ is mainly distributed in *indica*, a subspecies with high AC (> 20%), whereas *Wx*^*b*^ is widely found in *japonica*, a subspecies with low to intermediate AC (< 20%) [8, 11, 12]. Additional allelic variations have been identified from either natural or mutagenized varieties, such as *wx*, *Wx*^*mq*^, *Wx*^*hp*^, and *Wx*^*op*^ [13–17]. Two single nucleotide substitutions that cause missense base changes occurred in the *Wx*^*mq*^ coding region compared to the wild type allele (*Wx*^*b*^). The *Wx*^*mq*^ allele was first identified from Milky Queen, a *japonica* variety, and subsequently detected in other soft rice varieties, such as Kanto194, and Joiku436. Low AC (~ 10%) and high chalkiness in rice seed with *Wx*^*mq*^ may be due to the impaired enzyme activity of the GBSSI protein [17].

In recent decades, many minor QTLs non-allelic to *Wx* for AC, such as *qAC*, *qHAC, QAc*, *amy*, *ac,* and *dull*, have been detected throughout the whole rice genome [4, 6, 7, 18–27]. Many of these loci were found to genetically interact with the *Wx* gene. For instance, several *dull* genes were found to affect the splicing efficiency of *Wx*^*b*^, which results in a low AC and dull endosperm [28–30]. In addition, more genes such as *OsBP-5*, *OsEBP-89*, *OsbZip58* and *OsMADS7* were identified by molecular biological and reverse genetic methods to be involved in transcriptional or posttranscriptional regulation of rice *Wx* and fine control of AC [31–33]. Moreover, some genes that were initially associated with a floury endosperm or shrunken seed were verified to affect starch biosynthesis and subsequently influence the AC of rice [34–37]. Therefore, the AC might be controlled by multiple pathways.

During the grain filling stage, environmental temperature is another important factor influencing rice quality [2, 3]. AC and chalkiness are both hypersensitive to air temperature [26, 38]. High temperature (HT) could result in a severe reduction in the AC in many *japonica* varieties, while cool temperature causes an increase in the AC [2, 10, 26, 39]. A high variation in ACs was observed in several varieties when they were exposed to different temperatures. In some *japonica* varieties, such as Panda and Nato, the AC could increase by 21–41% under a cool temperature (18 °C) condition [10], which was a greater increase than that from additive effect of many minor loci on rice AC [4, 5, 19]. These findings implied that the effect of temperature on AC might be greater than that of many genetic factors in rice genome.

Epistasis and QTL-environment interactions make AC regulation more complex. Chromosome segment substitution lines (CSSLs) are ideal materials for dissecting complex traits. CSSLs contain a fairly uniform background from the recipient parent but distinct substituted chromosome segments from the donor parent in each line. Each substituted segment can be regarded as a single Mendelian factor and the epistatic effects among different segments can be break down. The whole genome of the CSSL population is considered valuable genetic material to precisely identify QTLs and genes [40]. The first CSSLs with a small population size (only 22 lines) were constructed in mice [40]. Using these CSSLs, several important QTLs related to obesity, behavior and sterol metabolism were detected [41]. Many CSSLs were then constructed in crops, such as rice, wheat, maize, etc. [42]. By using a set of CSSLs (39 lines) derived from a cross of the *indica* variety Kasalath and the *japonica* variety Koshihikari, three major QTLs controlling the Cd concentration were detected on chromosomes 3, 6 and 8, respectively, in rice genome [43]. Recently, a critical locus, *qGPC-10*, responsible for grain protein content was detected on chromosomes 10 with CSSLs (39 lines). *OsGluA2* was further confirmed as a candidate gene of *qGPC-10* by map-based cloning strategy [44]. As CSSLs can simplify complex genetic traits, they are very effective in studying QTL-environment interactions. For example, Liu et al. demonstrated QTL-environment interactions affecting panicle number with a set of CSSLs, and 9 QTLs for panicle number were identified in rice under different environments [45].

Previously, we constructed a set of CSSLs using the *indica* variety 9311 and the *japonica* variety Nipponbare (NIP) as donor and recipient, respectively [46]. Using these CSSLs, several QTLs for grain shape, hybrid sterility and rice quality have been detected [26, 46, 47]. In this study, we conducted a genome-wide survey for rice AC using these CSSLs (35 lines) under several environmental conditions, including growth chambers set at different temperatures and field conditions over several planting seasons. We detected a major QTL on chromosome 3, *qSAC3*, for moderately increasing AC under different environmental conditions. The logarithm of odds (LOD) score of *qSAC3* (3.144) was greater than the threshold of 2.97 (*p* = 0.05), and *qSAC3* from 9311 (*qSAC3*^*ind*^) could increase the rice AC in NIP under multiple environmental conditions. Furthermore, introducing *qSAC3*^*ind*^ into the soft rice Nangeng9108 with *Wx*^*mq*^ increased seed AC and reduced the chalky appearance significantly, thus improving the rice quality of Nangeng9108.

## Results

### AC of CSSLs and NIP vary under different environments

A set of CSSLs was used in this study. The CSSLs were constructed previously using the *japonica* variety NIP and the *indica* variety 9311 as recipient and donor, respectively [46]. These plant materials are likely appropriate to dissect effective loci for rice AC because both parental varieties have the same allele (*Wx*^*b*^) in the major locus *Wx*. To examine the AC variation under different environments, CSSLs and NIP at milky stage were grown under four different conditions (see details in Materials and Methods). In brief, rice seeds sown in June and in July and plants grown in the field were set as normal season (NS) and late season (LS) treatments, respectively, while the plants transferred to growth chambers with different temperatures after flowering were set as high temperature (HT, 35 °C/28 °C) and room temperature (RT, 28 °C/22 °C) treatments. Since air temperature is the most important environmental factor, we calculated the mean temperatures of these four given conditions (Table 1). They can be ranked in order of increasing average day-time and night-time temperatures as follows: LS (25.4 °C/16.5 °C) < RT (28 °C/22 °C) < NS (29.9 °C/23.7 °C) < HT (35 °C/28 °C). As we expected, the lowest mean value of AC was observed under the HT condition, and vice versa. These results were consistent with previous reports that rice AC can be reduced by HT, but increased by cool temperature [2, 10].

**Table 1:** Statistical analysis of ACs in Nipponbare and CSSLs observed under the four experimental conditions

Environmental condition	Average temperature (day/night)	Nipponbare		CSSLs			
		Means	SD	Means	SD	Min	Max
HT	35 °C/28 °C	13.08	0.34	13.69	1.61	9.92	16.76
RT	28 °C/22 °C	17.61	0.49	16.21	1.74	13.81	21.12
NS	29.9 °C/23.7 °C	18.14	0.16	18.69	1.97	13.07	22.06
LS	25.4 °C/16.5 °C	21.04	0.15	23.06	1.56	19.4	25.70

**Fig. 1:**
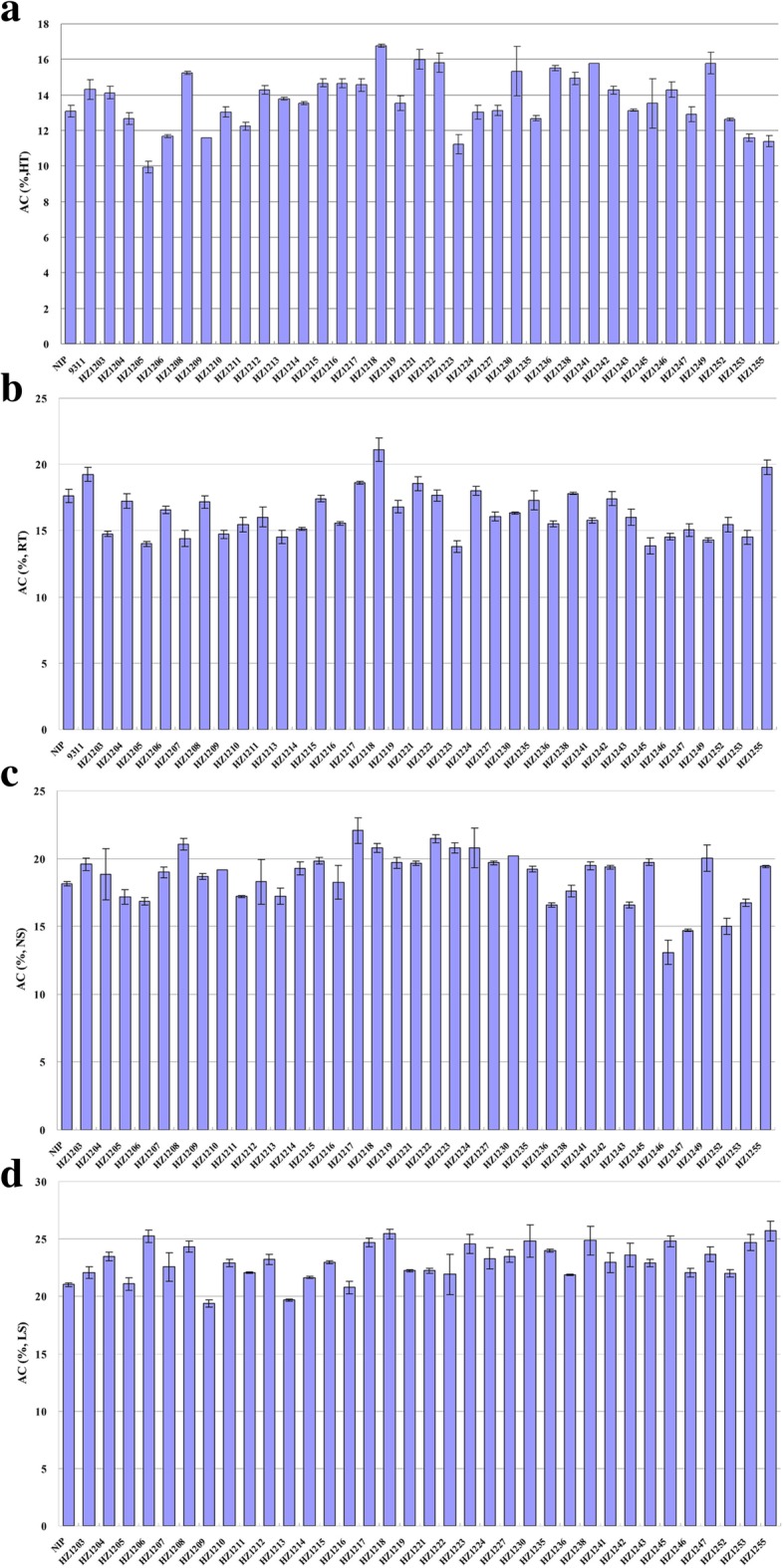
Amylose content of background parent Nipponbare and chromosome segment substitution lines under different conditions. Rice grains were filling at HT (high temperature, **a**), RT (room temperature, **b**), NS (normal season, **c**) and LS (late season, **d**) conditions. AC, Amylose content; NIP, Nipponbare

Furthermore, the AC variation among individual lines under different conditions was analyzed. Under HT condition, more than half of the CSSLs had a higher AC and ten lines had a lower AC than that of NIP (Fig. 1a). Among them, the AC of several CSSLs, such as HZ1218, HZ1221 and HZ1230, were significantly (*P* < 0.01) higher than that of NIP. In contrast, the AC of HZ1205, HZ1223 and HZ1255 were significantly (*P* < 0.01) lower than that of NIP (Fig. 1a). However, under RT condition, the rice AC of most CSSLs was lower than that of NIP, except for the four lines HZ1217, HZ1218, HZ1221 and HZ1255 (Fig. 1b). The AC of NIP was higher than the average AC of CSSLs at RT condition (Table 1), which was different from values observed in the other three environments.

Similar to what was observed at HT, the rice AC of most CSSLs was higher than that of NIP in the field experiment with NS condition (Fig. 1c). However, the line with the highest or lowest AC was different from that at HT. These results suggested that although several chromosome segments of 9311 could affect the AC of NIP under NS condition, the segments with the largest effect might be different from that under HT condition. Moreover, the average AC of the CSSLs was most dramatically increased under LS conditions compared to other conditions (Fig. 1d, Table 1). As the average temperature is lowest under the LS condition, this result suggests that 9311 is more sensitive to cool temperature in terms of the AC.

### *qSAC3* is responsible for rice AC under multiple environments

To facilitate further comparison of rice ACs under different environmental conditions, we defined the phenotype of the CSSLs as the difference of rice AC (D-value) between a CSSL and NIP (D-value = (AC_CSSL_-AC_NIP_)/ AC_NIP_). The D-value of each CSSL was obtained for each condition, and the D-value of NIP was zero for all tested conditions.

**Fig. 2:**
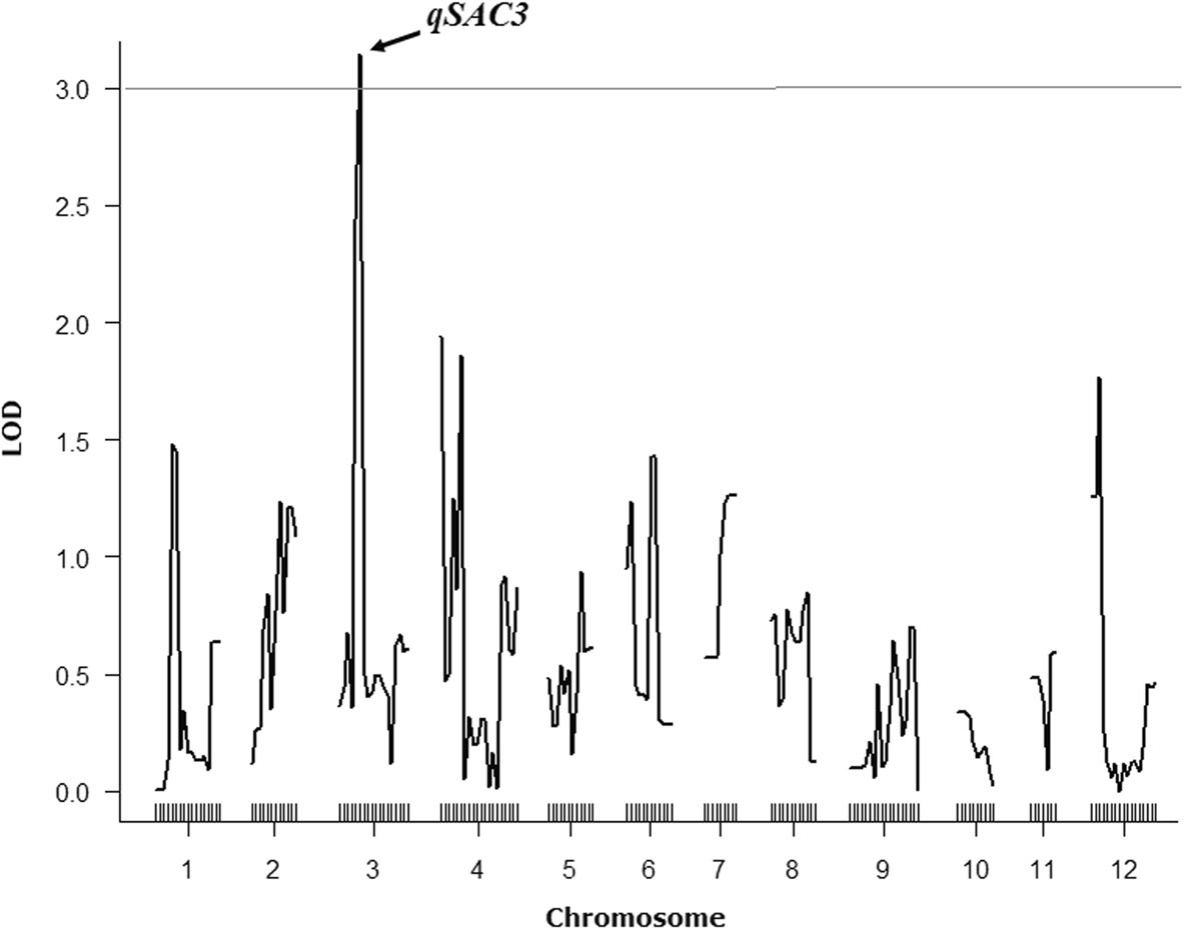
QTL analysis of rice AC at different environmental conditions. The gray line indicates the threshold of the LOD score, 2.97 (*p* = 0.05). The arrow indicates the location of *qSAC3*, which has an LOD score of 3.144

D-values from most of the CSSLs varied under the different environmental conditions, which suggested that many loci responsible for AC determination are not stable under varying environments. To identify the QTLs with stable effects on rice AC, a genome-wide survey was carried out by using the D-value as the phenotype. Eventually, only one locus above the threshold of 2.97 (*p* = 0.05) was detected in the rice genome (Fig. 2). The locus with the highest LOD score of 3.144 (*p* = 0.036) is located on chromosome 3 and is named *qSAC3* (locus with stable effect on **AC**). All the other peaks appearing in this survey were not significant, as their LOD were below the threshold (Fig. 2). These results indicate that, except for *qSAC3*, most substituted chromosome segments from the 9311 genome might have no effect on rice AC or have effects only under specific environmental/temperature conditions, and *qSAC3*^*ind*^ might be a valuable QTL affecting rice AC independent of the environment.

### The *qSAC3* from *Indica* 9311 increases rice AC of *japonica* NIP

**Fig. 3:**
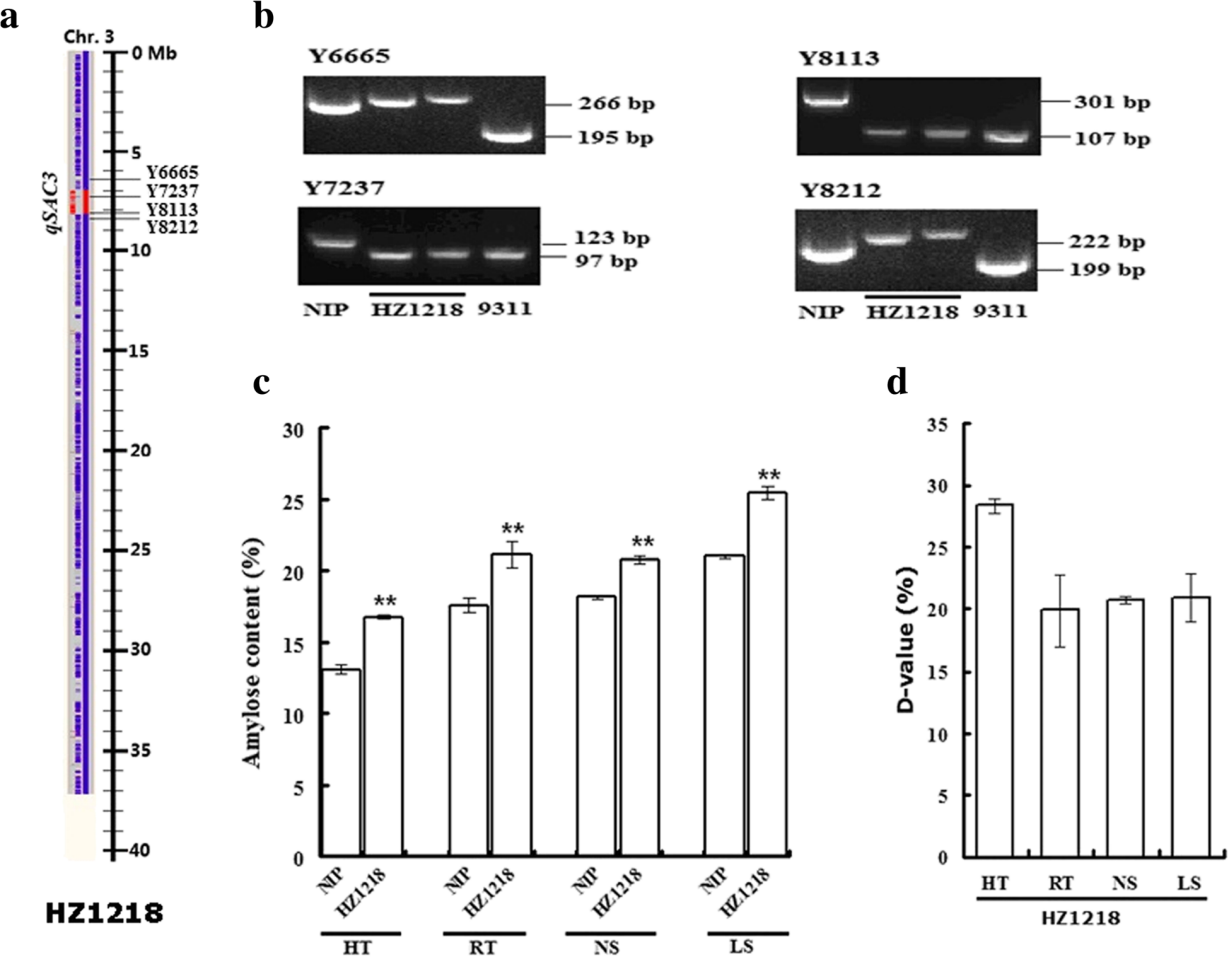
Genotype and phenotype of HZ1218. **a** Resequencing result of HZ1218. The red area indicates the substituted segment from 9311, and the blue area indicates background parent genotype. The short substituted segment on chromosome 3 in HZ1218 is the location of *qSAC3*. Molecular markers Y6665, Y7237, Y8113 and Y8212 are near the locus *qSAC3*. **b** PCR detection of HZ1218 by molecular markers Y6665, Y7237, Y8113 and Y8212. The results showed that the substituted segment on chromosome 3 contains Y7237 and Y8113 but not Y6665 and Y8212. **c** Rice AC of HZ1218 and NIP under different conditions. **d** Difference of rice AC (D-value = (AC_CSSL_-AC_NIP_)/AC_NIP_) between HZ1218 and NIP in different conditions. Significant differences were determined by Student’s t-test, *p*-value < 0.01(**)

Based on the resequencing results of CSSLs, *qSAC3* was located on a ~ 1.3 Mb segment from 6.9 to 8.2 Mb on chromosome 3 (Fig. 3a, Additional file 2: Figure S1). Molecular markers Y6665, Y7237, Y8113 and Y8212 were used to confirm the existence of a substituted segment on chromosome 3 (Fig. 3b, Additional file 1: Table S1). Six lines, HZ1211, HZ1212, HZ1213, HZ1214, HZ1218 and HZ1230, contain this chromosome segment (Additional file 2: Figure S1), and these lines were named CSSLs-*qSAC3* in this study. HZ1218 is the only line that had positive D-values under all environmental conditions (Fig. 3c-d). HZ1212, HZ1214 and HZ1230 had positive D-values in all environments except for the RT condition, while HZ1211 and HZ1213 had only one positive D-value at LS and HT, respectively (Additional file 2: Figure S1). These results indicate that some other loci might interact with *qSAC3*, affecting the AC in HZ1211, HZ1212, HZ1213, HZ1214 and HZ1230 under specific conditions. Therefore, HZ1218 is the best CSSL-*qSAC3* to evaluate the additive effect of the *qSAC3* from 9311 (*qSAC3*^*ind*^) on the AC.

Among the CSSL population, HZ1218 had the highest AC in the growth chambers (HT and RT) conditions and second and sixth highest AC in LS and NS field conditions, respectively (Fig. 1). The D-values of HZ1218 indicated that *qSAC3*^*ind*^ might have a large effect on rice AC in all tested experimental conditions (Fig. 3d). As shown in Fig. 3d, HZ1218 had the highest D-value (28.4%) at HT and similar D-values at RT (19.9%) and under NS (20.9%) and LS (20.9%) conditions. These results suggest that *qSAC3*^*ind*^ has a positive and stable effect on rice AC under different environmental conditions. Thus, *qSAC3*^*ind*^ could be used for quality improvement in some rice varieties, such as soft rice, which have an AC lower than that preferred by customers. In addition, although the AC of HZ1218 at HT (AC_HT-HZ1218_ = 16.76%) was lower than at RT, it was similar to that of NIP (AC_RT-NIP_ = 17.61%) at RT (Fig. 3c). Therefore, *qSAC3*^*ind*^ might have another important advantage in high-quality rice breeding in terms of heat resistance.

### Introducing *qSAC3*^*ind*^ into Nangeng9108 improves its quality

Nangeng9108 is a super rice variety in China with a high yield and eating quality. However, it has a dull endosperm, and thus, the polished rice has an ordinary appearance due to the *Wx*^*mq*^ allele, which is associated with a low AC (~ 10%). The ordinary appearance reduces the commercial value of Nangeng9108. It is proposed that the dull endosperm might be a consequence of low AC. We therefore tested whether *qSAC3*^*ind*^ could be used to increase AC and subsequently improve the appearance of Nangeng9108.

**Fig. 4:**
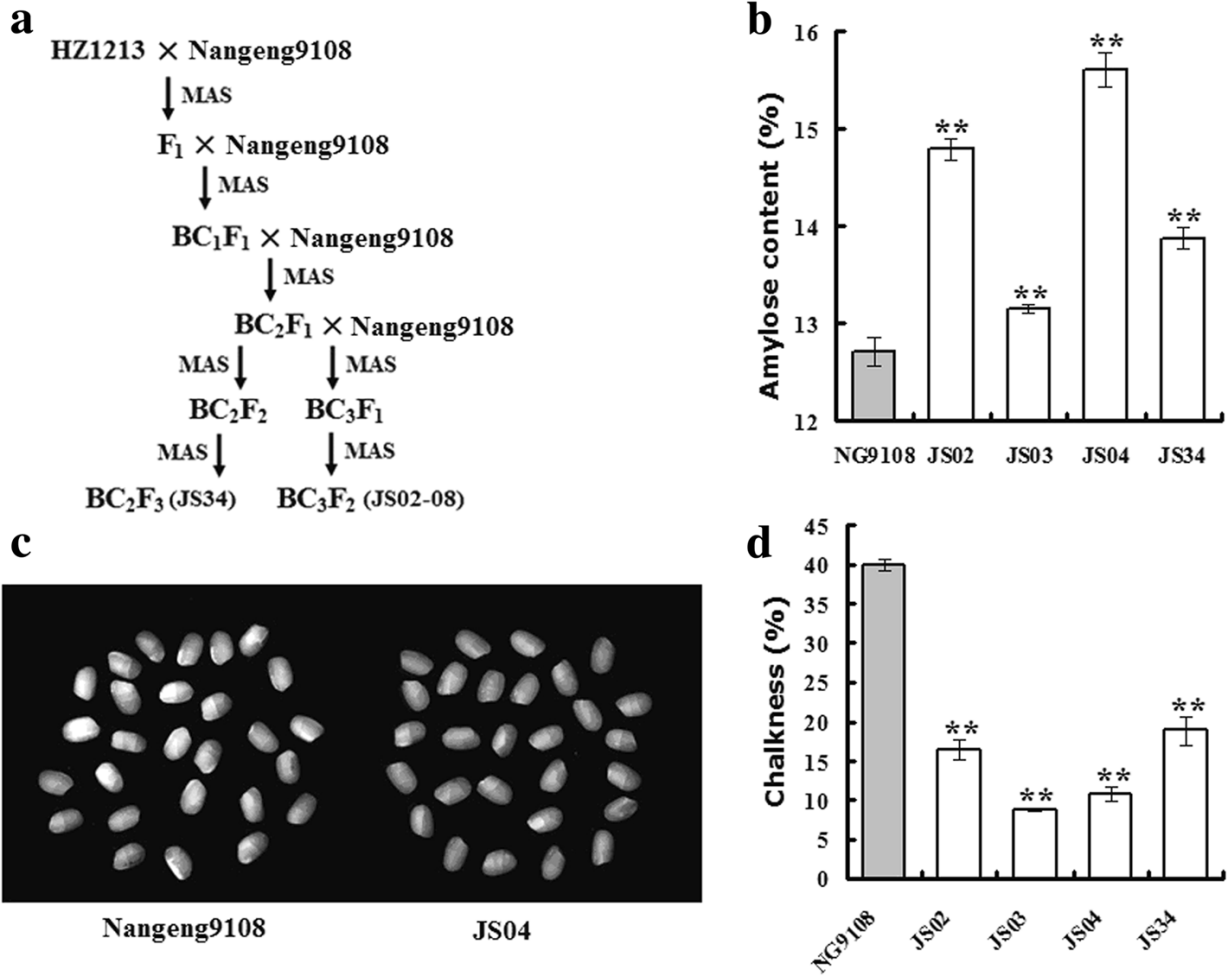
Introduction of *qSAC3*^*ind*^ into Nangeng9108 improves rice quality. (**a**) Generation of NILs-*qSAC3* (JS02–08 and JS34) by crossing Nangeng9108 with the CSSL HZ1213. **b** AC of Nangeng9108 (NG9108) and NILs-*qSAC3* (JS02, 03, 04 and 34). **c** Polished rice of Nangeng9108 and JS04. **d** Chalkiness of Nangeng9108 (NG9108) and NILs-*qSAC3* (JS02, 03, 04 and 34). Significant differences were determined by Student’s t-test, *p*-value < 0.01(**)

A single segment substituted line (HZ1213, Additional file 3: Figure S2) containing *qSAC3*^*ind*^ on chromosome 3 was used as the donor for the cross with Nangeng9108. Eight near-isogenic lines (NILs-*qSAC3*^*ind*^) were identified by marker assisted selection, including the F_2_ progeny of the third backcross lines, JS02, JS03, JS04, JS05, JS06, JS07, JS08 (BC_3_F_2_), and the F_3_ progeny of the second backcross line, JS34 (BC_2_F_3_) (Fig. 4a). These plants showed similar growth phenotypes to that of the receptor line Nangeng9108. Four lines (JS02, JS03, JS04 and JS34) were selected for the rice quality assay. The AC of NILs-*qSAC3*^*ind*^ (13.2–15.6%) was significantly higher than that of Nangeng9108 (12.7%, Fig. 4b). The appearance of NILs-*qSAC3*^*ind*^ was also improved compared to that of Nangeng9108. Both the transparence and chalkiness of the polished rice from NILs-*qSAC3*^*ind*^ (transparence level was 3 and chalkiness score was 8.8–18.9%) were significantly lower than that of Nangeng9108 (transparence level was 5 and chalkiness score was 40.0%, Fig. 4c-d). These results implied that introduction of *qSAC3*^*ind*^ into Nangeng9108 could increase the rice AC and ameliorate the dull endosperm caused by the *Wx*^*mq*^ allele.

In addition to AC, we also evaluated the effect of *qSA*C3 on rice GT and GC by using another segregated population (~ 240 plants) generated from heterozygous plants of NILs-*qSAC3*^*ind*^ in the background of Nangeng9108. As expected, rice AC and chalkiness were cosegregated with *qSAC3*. Most plants with *qSAC3*^*ind*^ were found to have a higher rice AC but lower chalkiness than the plants with the allele *qSAC3*^*jap*^ (Additional file 4: Figure S3a-b). However, no significant difference was found in rice GT (negatively related to the alkali spreading value) and GC between rice plants with *qSAC3*^*ind*^ and *qSAC3*^*jap*^ (Additional file 4: Figure S3c-d). These results indicated that *qSAC3*, a locus for rice AC, might have no/little effect on rice GT and GC. Thus, *qSAC3*^*ind*^ might be a good genetic resource to increase the AC and appearance quality of soft rice varieties, such as Nangeng9108, Milky Queen, Kanto194, and Joiku436.

## Discussion

With the development of a global economy, the demands for rice quality are increasing. The AC, as a major physicochemical property of starch, could significantly influence rice eating and cooking quality [1]. Elucidating the mechanisms of AC determination and regulation is essential for rice quality improvement [4, 5]. However, the inheritance of AC is complex. More importantly, the AC itself is very sensitive to environment [2, 3], which makes high-quality rice breeding and expansion more difficult. To identify effective loci to compensate for the AC variation based on environmental conditions, a set of CSSLs from a cross between the *japonica* variety NIP and the *indica* variety 9311 [46] was used for the survey in this study. These CSSLs are good materials for dissecting the QTLs-environment interaction of rice AC because both parental varieties have the same *Wx*^b^ allele and a similar AC under normal growth conditions but different ACs in response to temperature stress [12, 26]. High variation in rice ACs in most CSSLs was observed under different growth conditions in our survey, which implied that there might be some loci responsible for rice AC under specific conditions. However, only one locus (*qSAC3*) was identified above the threshold across all tested conditions. The low LOD score of other regions suggests that most loci responsible for AC in the rice genome might not be effective in multiple environments. The AC of CSSLs-*qSAC3*^ind^ implied that the *indica* allele from 9311 has a positive effect on the *japonica* rice NIP. This positive effect was confirmed by several NILs-*qSAC3*^ind^ in the background of another *japonica* variety, Nangeng9108. The results from a segregated population (~ 240 plants) further indicated that *qSAC3*^ind^ had a significant effect on AC but had no/little effect on GC and GT in *japonica* rice. Thus, the positive effect of *qSAC3*^ind^ on the AC of *japonica* rice is credible, and the locus could be used for breeding high-quality rice with wide adaptability. Two large segregated populations (> 5000 plants) were generated from CSSLs-*qSAC3*^ind^ and NILs-*qSAC3*^ind^ crossed with their recurrent parents NIP and Nangeng9108, respectively, and will be used for fine-mapping of *qSAC3* in the future.

A dull endosperm impairs the appearance of rice and thus reduces its commercial value. Many mutations that occur in the coding region of *Wx*^*b*^ allele, such as *Wx*^*mq*^, and *Wx*^*hp*^, could reduce the enzyme activity of the GBSSI protein and thus cause a low AC and dull endosperm in the varieties with such *Wx* alleles [13–17]*.* Furthermore, many *dull* rice mutants are found to show a low AC (6–12%) and dull endosperm [28–30]. However, this phenotype in *dull* mutants is not caused by low GBSSI activity but instead by low *Wx*^*b*^ transcript levels [30]. Therefore, in most of cases, a dull endosperm is due to an impaired GBSSI level or activity. In our study, we showed that *qSAC3* has an additive effect on *Wx*^*b*^. Introducing of *qSAC3*^*ind*^ into Nangeng9108, a soft rice variety with *Wx*^*mq*^, could significantly increase the AC and decrease the chalkiness of the variety. The molecular mechanism of the *qSAC3* locus in AC regulation remains unknown at this stage. It will be interesting to further test whether *qSAC3* could genetically interact with the *Wx* gene, such as exploring whether the locus has any effects on the regulation of *Wx*^*b*^ expression at the transcriptional or posttranscriptional level or modification of GBSSI activity at posttranslational level. Moreover, the additive effect of *qSAC3* on other mutant alleles of *Wx*^*b*^, such as *Wx*^*hp*^, also needs to be investigated in the future. In addition to *Wx*^*b*^, another main *Wx* allele, *Wx*^*a*^, is widespread in nature. However, few studies have investigated the regulation of *Wx*^*a*^ under different growth conditions. Therefore, inspecting the genetic effects of *qSAC3* in the *Wx*^*a*^ background will be another interesting study.

HT at the grain filling stage can harm rice grain quality by significantly reducing the AC in many rice varieties. To uncover the molecular mechanism of HT effects on rice AC, we carried out a genome-wide survey using the same set of CSSLs, previously. Several QTLs, *qHAC4*, *qHAC8a* and *qHAC8b*, which can stabilize the rice AC at HT were detected and further verified to be involved in the processing of mature *Wx* mRNA under HT conditions [26]. We speculate that there might be some important factors involved in raising the splicing efficiency of *Wx* pre-mRNA in these loci. Meanwhile, an HT responsive gene, *OsMADS7*, was isolated in another previous study [32]. Suppression of its expression in rice endosperm could also stabilize rice AC at HT. More mature transcripts of *Wx*^*b*^ were detected in the endosperm of *OsMADS7* RNAi seeds than in wild type seeds [32]. These results indicate that posttranscriptional regulation of *Wx*^*b*^ is a critical mechanism for maintaining AC stability at HT. Here, we report that the *qSAC3*^*ind*^ could increase rice ACs at multiple conditions. HZ1218, a CSSL containing *qSAC3*^*ind*^, had the highest D-value for rice ACs at HT. HZ1218 had a similar AC (16.76%) at HT to that of NIP (17.61%) at RT, which implied that introducing the *qSAC3*^*ind*^ into *japonica* cultivars could generate a suitable AC at HT. Although the mechanism of the *qSAC3* modulation of rice AC remains unknown and the genetic interaction among *qHACs*, *OsMADS7* and *qSAC3* need to be investigated further, comprehensive utilization of these loci and genes might be very valuable for breeding heat stable rice varieties with high quality.

In the rice genome, numerous QTLs responsible for AC have been detected in recent decades. Although it is widely recognized that the rice AC is determined by one major locus, *Wx*, and several minor loci, some researchers are still trying to uncover other major QTLs for AC. In addition to *Wx*, Huang et al. [21] detected another major locus responsible for rice AC on chromosome 3 by using a RIL population derived from a cross between CT9993 (*japonica*) and KDML105 (*indica*). The major QTL was located in a large region (~ 30.3 cM) on chromosome 3 which might contain the candidate segment of *qSAC3* based on the position of linked markers. Moreover, in the same region, another QTL for AC, ac3.1, was also detected between markers RM22 (~ 1.5 Mb) and RM7 (~ 9.9 Mb) by Swamy et al. [22] from a cross between cultivar Swarna (*O.sativa indica*) and wild rice IRGC81848 (*O.nivara*). Such above QTLs responsible for AC from the *indica* rice KDML105 and Swarna also show positive effects on *japonica* rice in terms of AC. Thus, considering of the similar location and effect of these QTLs, we proposed that *qSAC3*, ac3.1 and the major locus detected by Huang might be the same QTL for rice AC and that the allele from *indica* rice might show a positive effect in *japonica* rice.

## Conclusions

*qSAC3* might be a major locus responsible for AC modulation, and its *indica* rice allele could increase the AC of *japonica* rice in the background of *Wx*^*b*^ under a variety of conditions. Introducing *qSAC3*^*ind*^ into the soft rice Nangeng9108 with the *Wx*^*mq*^ allele could moderately upregulate rice AC and significantly improve the appearance of polished rice. These results suggest that *qSAC3* might be a valuable locus for high-quality rice breeding under multiple environments, especially HT conditions.

## Methods

### Growth conditions of CSSLs

The original CSSLs from Yangzhou University contain 57 lines. Their genotype was confirmed by molecular markers and high-throughput resequencing. In this study, 35 lines were used and they had 89 substituted segments derived from the *indica* variety 9311, which covered approximately 82.8% of the whole rice genome [46].

Seeds of CSSLs were sown in June, and plants were transplanted in July 2012 in Haining, Zhejiang Province, which was considered the field experiment under normal season (NS) treatment. Fifty days after sowing, eight plants of each line were carefully transplanted into two pots. Three days after flowering, the plants were transferred either into a 35 °C, 12 h light/28 °C, 12 h dark chamber for high temperature (HT) or into a 28 °C, 12 h light/22 °C, 12 h dark chamber for room temperature (RT) treatment [26]. Another field experiment with the CSSLs was conducted in Hangzhou, Zhejiang Province. Rice seeds were sown in July, and plants were transplanted in August, which was considered the late season (LS) treatment in our study. Approximately 40 days after heading, ripe seeds of CSSLs and the parent variety NIP were harvested from the four environmental treatment groups: HT and RT, applied via chambers, and NS and LS, applied via experimental field studies, in Haining and Hangzhou, respectively.

NILs of *qSAC3* were developed with the *japonica* variety Nangeng9108 as a receptor in Nanjing, Jiangsu Province from 2013 to 2017. Seeds of NILs (JS02–08, JS34) were sown in June, and plants were transplanted in July 2017 in Nanjing, Jiangsu Province. Matured seeds of NILs were harvested in November 2017 and then were used for quality assay. Seeds of a segregated population (~ 240 plants generated from NILs crossed with Nangeng9108) were sown in June. Ripe seeds were harvested in November 2018 and then were used for quality analysis.

### Determination of rice quality

Rice starch was purified from polished rice using the alkaline protease method [48, 49] and was then used to measure AC and gel consistency (GC). AC was measured by an iodine colorimetric method [1] with slightly modification [26]. Ten milligrams of rice starch was digested in 1 M NaOH at room temperature (25 °C) for 16–18 h. After ten-fold dilution with distilled water, digestive juices were incubated with 100 mM acetic acid and 200 mM of 0.2% (w/v) I2 and 2% (w/v) KI at room temperature (25 °C) for 15 min. Absorbance of the reaction solution was measured at a wavelength of 720 nm according to the Standards of the Agricultural Department, People’s Republic of China (2008). A standard curve was generated from the analysis of the results of the standard samples obtained from the China National Rice Research Institute. At least three repeats were conducted for each line.

GC was measured according to the method of Cagampang et al. [50]. One hundred milligrams of rice starch was digested in 2 ml KOH (0.2 M) in a bath of boiling water for 8 min. After cooling at room temperature (25 °C) for 5 min, digestive juices were immersed in ice water for 20 min and then placed horizontally for an hour. Finally, the length of the gel was recorded as the value of the GC, and three repeats were conducted for each line.

The gelatinization temperature (GT) was represented by the alkali spreading value measured according to the method of Little et al. [51]. Ten polished rice were immersed in 10 ml KOH (1.7%) at room temperature (25 °C). After 23 h, the spreading value of each line was recorded based on the degree of the endosperm corrosion, and three repeats were conducted for each line.

The transparence and chalkiness of polished rice with *Wx*^*mq*^ was analyzed quantitatively for dullness of the endosperm by an SC-I test system for crop seeds (Wseen, China). At least 100 plump rice seeds were tested for each line.

### QTL analysis of rice AC

The genotype of the CSSLs was reconstructed based on the resequencing results [46]. The difference in AC〔D-value = (AC_CSSL_-AC_NIP_)/ AC_NIP_〕between CSSLs and NIP was used as trait. Therefore, each CSSL had a D-value for each environmental condition and the D-values of NIP under each environmental condition were set as zero. QTL mapping was performed using the R/qtl package [52]. LOD scores were calculated with a single-QTL model using the function “scanone” with a Haley-Knott regression method [53]. The LOD score significance threshold was established using 1000 permutations. A *p* value of 0.05 was selected and the corresponding threshold of LOD score was 2.97 in our study. Substituted segments with similar D-values under all tested conditions might have high LOD scores. Thus, loci detected in this QTL analysis would have a stable and environment-independent effect on rice AC.

### Construction of near-isogenic lines

Nangeng9108, a *japonica* super rice variety with a high yield in China, was developed from a cross between the *japonica* cultivar Kanto194 and Wuxianggeng14. Nangeng9108 has a high eating and cooking quality and is preferred by many people in China. However, the general appearance of polished rice due to the dull endosperm reduces the commercial value of Nangeng9108. To introduce the major locus into Nangeng9108, it was crossed with line HZ1213, a single segment substitution line (Additional file 3: Figure S2) containing *qSAC3*^*ind*^ locus. F_1_ plants were backcrossed with Nangeng9108 several times. In each generation, rice plants were identified with the markers-assisted selection method. Rice seeds of Nangeng9108 and near-isogenic lines of *qSAC3* (NILs-*qSAC3*) were sown in May and rice plants were transplanted in June 2017 in Nanjing, Jiangsu Province. Approximately sixty days after heading, ripe seeds were harvested for the rice quality assay.

## Additional files

Additional file 1: **Table S1** Primer sequences and products of polymorphic bands for the developed markers (DOC 27 kb)

Additional file 2: **Figure S1** Resequencing result (chromosome 3) and D-values of CSSLs which containing the *qSAC3*. Blue bars and red bars represent the background of Nipponbare and substituted chromosome segments from 9311, respectively. The D-values of CSSLs showed positive (+, > 0) or negative (−, < 0) effects on rice AC, and NS represented no test result. (JPG 148 kb)

Additional file 3: **Figure S2** Resequencing results of CSSL HZ1218 and HZ1213. Blue bars and red bars represent the background of Nipponbare and substituted chromosome segments from 9311, respectively. HZ1213 was selected to introduce the *qSAC3*^*ind*^ into the *japonica* variety Nangeng9108, because it only takes a single substituted segment on chromosome 3. (JPG 155 kb)

Additional file 4: **Figure S3** Rice quality of plants with *qSAC3*^*ind*^ and *qSAC3*^*jap*^. It is shown that rice plants with *qSAC3*^*ind*^ and *qSAC3*^*jap*^ have significant differences in rice AC (a) and chalkiness (b), but not in GT (c, gelatinization temperature represented with alkali spreading value) and GC (d, gel consistency). (JPG 1521 kb)

## Data Availability

The datasets generated and analyzed during the current study are available from the corresponding author (Y. Zhu) on reasonable request.

## Abbreviations

AC: Amylose content
CSSLs: Chromosome segment substitution lines
D-value: The difference in rice AC between a CSSL and NIP
GC: Gel consistency
GT: Gelatinization temperature
HT: High temperature
LOD: Logarithm of odds
LS: Late season
NILs: Near-isogenic lines
NIP: Nipponbare
NS: Normal season
*qSAC3*^*ind*^: *qSAC3* from the *indica* variety 9311
*qSAC3*^*jap*^: *qSAC3* from *japonica* variety
QTLs: Quantitative trait loci
RT: Room temperature
