# Supplementary material for: The qSAC3 locus from indica rice effectively increases amylose content under a variety of conditions
Source: BMC Plant Biol. 2019 Jun 24;19:275. doi: 10.1186/s12870-019-1860-5 (PMC6591921; doi:10.1186/s12870-019-1860-5)
Supplement: Supplementary file 1 — Table S1 Primer sequences and products of polymorphic bands for the developed markers (DOC 27 kb) [file 12870_2019_1860_MOESM1_ESM.doc]

**Table S1** Primer sequences and products of polymorphic bands for the developed markers

| Primer | Sequence(5'-3') | | Products(bp) | |
| --- | --- | --- | --- | --- |
|  | Forward | Reverse | Nipponbare | 9311 |
| Y6665 | GGAGATGGACAATGCTGAAA | GCACGAGATCTAGTACTCAT | 266 | 195 |
| Y7237 | CTAGAACCATTACCAGTCCA | CTAAAAAGTCAACGGCGTCA | 123 | 97 |
| Y8113 | TTCACAATCTCCCCTCAGTT | TTGAACATGTGGAGGTAGCA | 301 | 107 |
| Y8212 | CACCGAACAGAGCCTAAGTT | GATTACCGGGTGGGATTAGT | 222 | 199 |
