# Supplementary figures and images for: The qSAC3 locus from indica rice effectively increases amylose content under a variety of conditions
Source: BMC Plant Biol. 2019 Jun 24;19:275. doi: 10.1186/s12870-019-1860-5 (PMC6591921; doi:10.1186/s12870-019-1860-5)

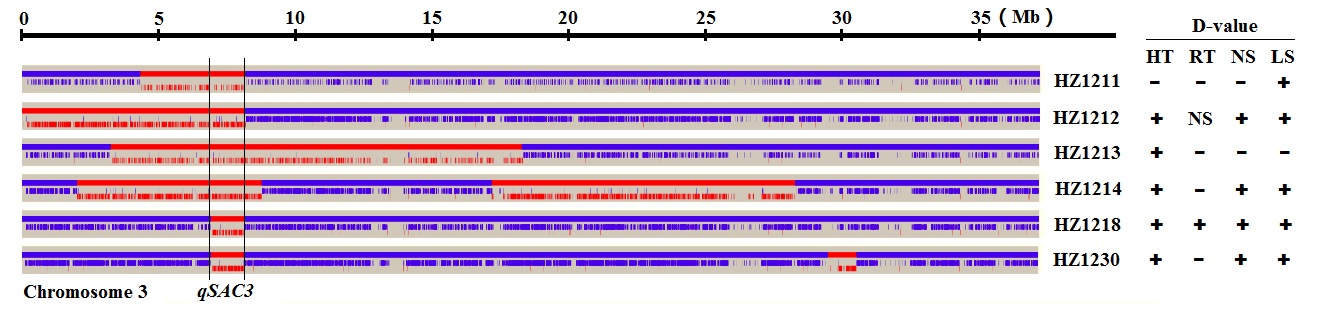

Supplement: Supplementary file 2 — Figure S1 Resequencing result (chromosome 3) and D-values of CSSLs which containing the qSAC3. Blue bars and red bars represent the background of Nipponbare and substituted chromosome segments from 9311, respectively. The D-values of CSSLs showed positive (+, > 0) or negative (−, < 0) effects on rice AC, and NS represented no test result. (JPG 148 kb) [file 12870_2019_1860_MOESM2_ESM.jpg]

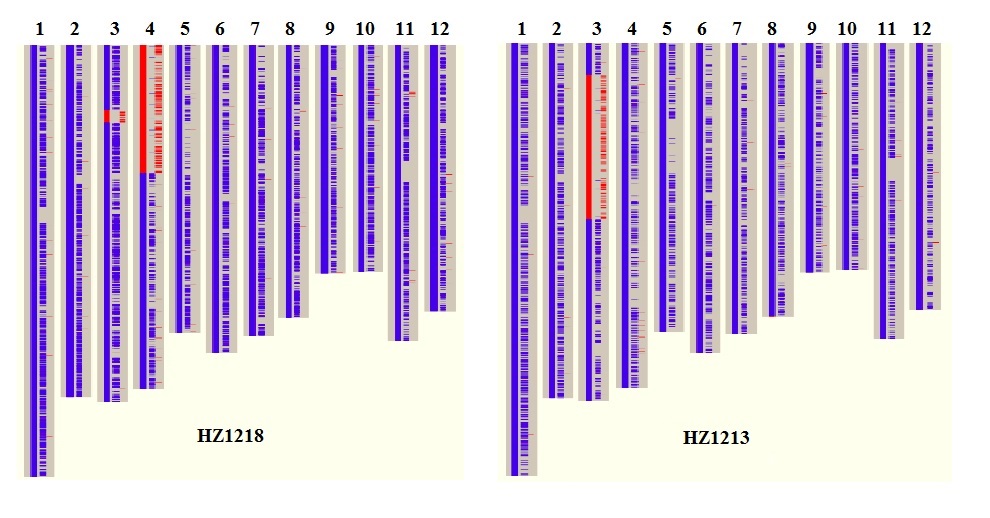

Supplement: Supplementary file 3 — Figure S2 Resequencing results of CSSL HZ1218 and HZ1213. Blue bars and red bars represent the background of Nipponbare and substituted chromosome segments from 9311, respectively. HZ1213 was selected to introduce the qSAC3ind into the japonica variety Nangeng9108, because it only takes a single substituted segment on chromosome 3. (JPG 155 kb) [file 12870_2019_1860_MOESM3_ESM.jpg]

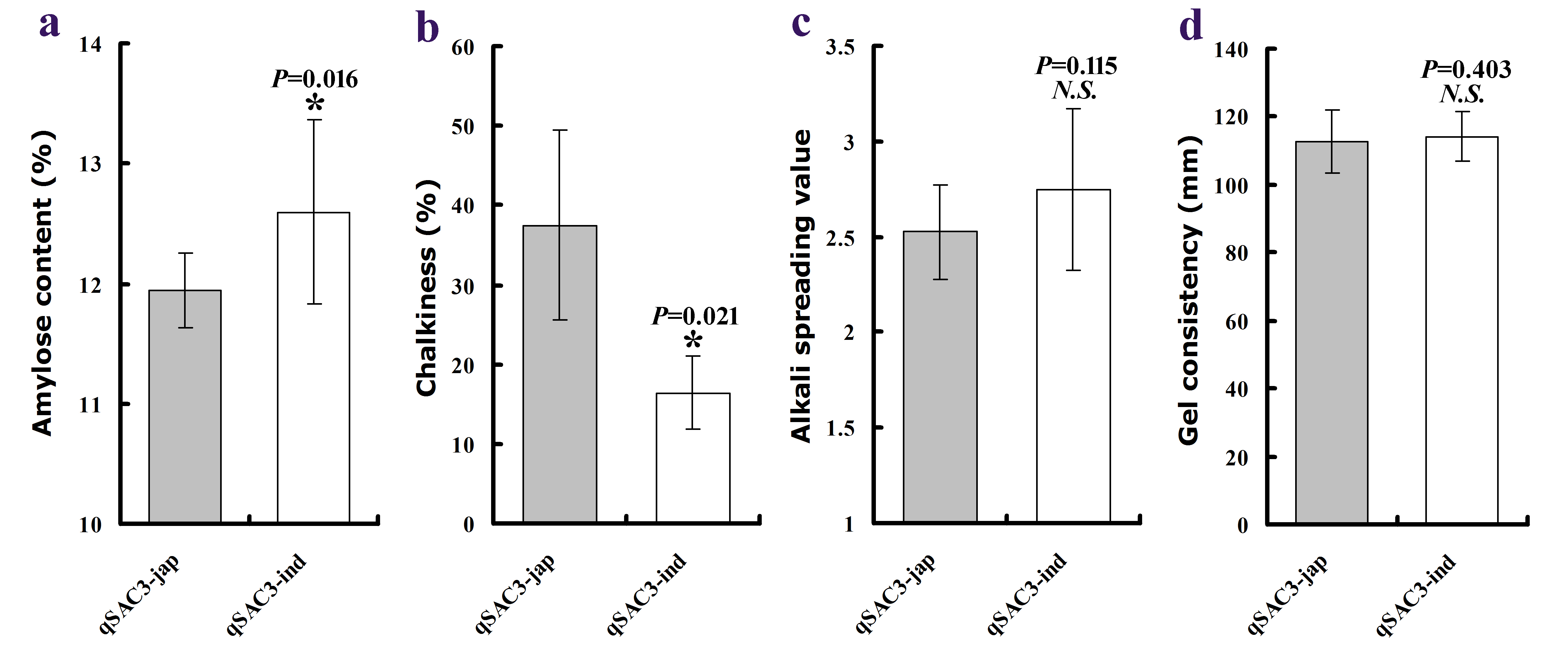

Supplement: Supplementary file 4 — Figure S3 Rice quality of plants with qSAC3ind and qSAC3jap. It is shown that rice plants with qSAC3ind and qSAC3jap have significant differences in rice AC (a) and chalkiness (b), but not in GT (c, gelatinization temperature represented with alkali spreading value) and GC (d, gel consistency). (JPG 1521 kb) [file 12870_2019_1860_MOESM4_ESM.jpg]
